# Mathematical Model for Growth Regulation of Fission Yeast *Schizosaccharomyces pombe*

**DOI:** 10.1371/journal.pone.0049675

**Published:** 2012-11-27

**Authors:** Luca Cerone, Béla Novák, Zoltán Neufeld

**Affiliations:** 1 School of Mathematical Sciences and Complex and Adaptive Systems Laboratory, University College Dublin, Dublin, Ireland; 2 Department of Biochemistry, University of Oxford, Oxford, United Kingdom; 3 School of Mathematics and Physics, University of Queensland, Brisbane, Australia; University of Cambridge, United Kingdom

## Abstract

Regulation of polarised cell growth is essential for many cellular processes including spatial coordination of cell morphology changes during the division cycle. We present a mathematical model of the core mechanism responsible for the regulation of polarised growth dynamics during the fission yeast cell cycle. The model is based on the competition of growth zones localised at the cell tips for a common substrate distributed uniformly in the cytosol. We analyse the bifurcations in this model as the cell length increases, and show that the growth activation dynamics provides an explanation for the new-end take-off (NETO) as a saddle-node bifurcation at which the cell sharply switches from monopolar to bipolar growth. We study the parameter sensitivity of the bifurcation diagram and relate qualitative changes of the growth pattern, e.g. delayed or absent NETO, to previously observed mutant phenotypes. We investigate the effects of imperfect asymmetric cell division, and show that this leads to distinct growth patterns that provide experimentally testable predictions for validating the presented competitive growth zone activation model. Finally we discuss extension of the model for describing mutant cells with more than two growth zones.

## Introduction

One of the major tasks that a eukaryotic cell faces during its life cycle is spatial organization of its components. The correct organization of the cellular components is fundamental not only for the defining and maintaining the correct shape, but also for cellular functions, such as movement, growth and the spatial coordination of cell division. To accomplish these goals, cells have developed complex strategies that allow them to recognize the different parts of the cell, sense cell length and detect the axis of growth. These strategies often involve the polarized distribution of ad hoc proteins within the cell and, although the exact mechanisms are different in different cell types, some common strategies have been identified [1]–[3].

The fission yeast *Schizosaccharomyces pombe* has proved to be a good model to understand the basic mechanisms involved in cell polarization and morphogenesis due to its particular shape and characteristic pattern of growth. *S. pombe* cells are cylindrical, grow by tip extension and divide by medial fission. Immediately after the division the fission yeast is about 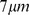 long; during its life it doubles its length till reaching approximately 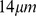 before division, while keeping its diameter of 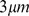 constant [1], [4]. Upon reaching the division length the cell stops growing and enters into mitosis at the completion of which the cell divides into two daughter cells of approximately the same length [4]. As a result of the division each of the new born cells inherits a tip that pre-existed the division (*old end*) and a newly formed tip deriving from the septation (*new end*). Immediately after division the growth proceeds in a monopolar fashion by extending only the old tip; in early G2 phase, between 0.3–0.4 of the cell cycle, the new end starts growing and the growth proceeds on both tips; at around 0.75 of the cell cycle it stops growing and enters M phase for cytokinesis and septation [2], [4], [5]. The switch from monopolar to bipolar growth is known as *New End Take-Off* (NETO) and it is a characteristic feature of the fission yeast cell cycle.

The isolation and characterisation of morphology mutants provided invaluable insight into the regulation of polarized growth. The components involved in regulation of polarized growth in fission yeast can be classified into one of the following two processes. Some components are responsible for marking the appropriate regions of the cortex for growth and while other ones are redirecting the growth machinery towards these marked locations [5]. Orb mutants are defective in establishment of polarized growth therefore they lose the characteristic rod shape and they become round [6]. Cytoplasmic microtubules running parallel to the long axis of the cell are responsible to deliver *land-mark* proteins tea1, tea4 and tea2 to the cell tips [1], [3]. Tea mutants fail to activate the growth at the new cell end therefore they show monopolar growth during their cycle. Tea mutants got their name because a small fraction of the population activates growth in the middle of the cell producing T-shaped cells [6]. The phenotype of for3 mutants are different. After division one of the daughter cells grows from the old end and shows no NETO, while the other starts growing from both ends almost immediately undergoing NETO very prematurely [7].

At cell tips, tea1 and tea4 recruit further polarity factors like for3 to the cell tips that give rise to a protein complex that mediates the actin assembly and membrane trafficking [2]. tea1 tea4 and for3 are at the heart of a complex called the polarisome which triggers to the formation of actin filaments and patches at the cell tips. Actin patches are associated with the sites of active growth and contribute to the correct trafficking of the cargo vesicles containing the material required for tips extension [1], [2], [5]. The activity of the tea1/tea4/for3p complex is regulated by other proteins that contribute to cell polarity like pom1 [8], cdc42 [9] and bud6 [10]. pom1 mutants cannot keep the long cellular axis straight but they are also defective in selecting and turning on growth at the right cell tip [8].

Despite of the increasing knowledge of the molecular details regulating cell polarity, the mechanism of growth zone activation is still mysterious. It is still not well understood how fission yeast succeeds in activating bipolar growth upon reaching a threshold cell length and how different mutants are defective in this mechanism. A potential reaction-diffusion mechanism has been proposed in which the two growth zones are competing autocatalytically for the same cytoplasmic ‘activator’ [11]. The essence of this mechanism has recently received some experimental confirmation by detecting competition of growing tips for active Cdc42 or its regulators [9]. Since competition was accompanied by dynamic oscillation of active Cdc42 at cell poles, the mathematical model has been extended by time-delayed negative feedback loop [9].

In this work we revisit the problem of length-dependent activation of bipolar growth by considering the original mathematical model for the activation of tip growth in fission yeast [11] and reduce it to the essential ingredients necessary for producing the qualitative behaviour consistent with NETO. We further simplify the mechanism by taking into account the spatial localization of components bound to the growth zones and assuming rapid diffusion of the cytoplasmic activator thereby reducing the model to ordinary nonlinear differential equations. This simplification allows us to use the powerful tools of nonlinear dynamical systems like bifurcation diagrams. We show how competition of the growth machinery at cell tips for a common substrate (‘activator’) can implement a length-sensing mechanism and allows the switch from monopolar to bipolar growth upon reaching a threshold cell length. We link modifications in the parameters of the model to observed mutant phenotypes. We also investigate the role of cellular asymmetry and its effects on NETO. In the final section we study the case in which growth is not restricted only to cell tips, and we allow for growth zone activation on the lateral cortex.

## The Model

Although positioning of growth zones is an important step in morphogenesis, we focus our attention only on activation of established growth zones. Therefore we investigate a model of the growth zone activation mechanism that describes the main characteristics of the growth patterns observed in wild type and mutant fission yeast cells [11]. In this model the growth zones are established at the two cell tips by a complex of proteins (tea1, tea4, for3, etc.), called *polarisome*. The co-localization of polarisome components at cell tips provide the cell with the potential for growth zone activation. The activation of the growth zone is limited by a component 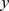 and without this component growth zones stay inactive and labelled by 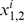. The established growth zones compete for this limiting component (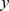), which is a freely diffusible substrate of their activation. The validity of the model is rather independent from the biochemical nature of this component, it is rather determined by the assumption that growth zone activation has a rate-limiting step controlled by a single component. Still, the recent work by Das et al suggests that the limiting component for growth zone activation is Cdc42 or one of its regulators [9].

**Figure 1:**
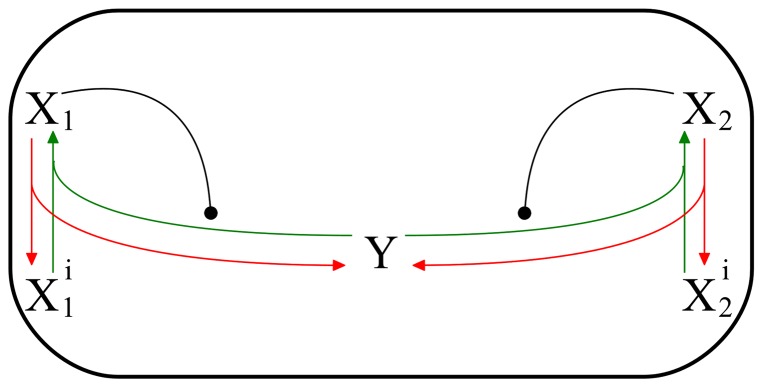
Schematic diagram of the model: 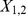 are localised to the tips and 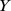 diffuses quickly in the cytosol. The inactive forms 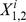 are activated autocatalytically by forming a localised complex with 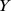 that is inactivated when 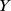 dissociates from 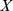 and returns to the cytosol.



The model assumes that activation of the growth zone depends on binding of this limiting component (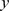) to the inactive growth zone. While the sum of the inactive (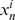) and active (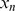) forms of growth zone at each cell ends are assumed to be constant 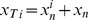, 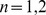, the total amount of 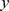 is proportional to the length of the cell (growth dependent). The activation of growth zone is assumed to be an auto-catalytic process, that is the active fraction of the growth zone (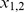) enhances the rate of activation of the inactive fraction (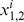). The growth zone activation is a dynamic process which is reversed by the spontaneous inactivation of the active forms into inactive ones (Figure 1). This activation-inactivation cycle can be described by the following system of differential equations:

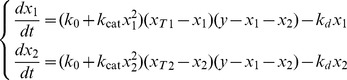
(1)where 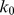 is the basal activation rate, 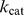 is the coefficient of the auto-catalytic activation and 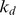 is the dissociation coefficient (reflecting inactivation). Since the total amount of 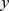 is conserved, 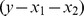 represents the amount of the limiting component that is not bound to the growth zones. The 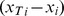 terms represent the inactive fraction of a particular growth zone that can be activated. In this model we assume that the timescale of diffusion of the substrate over the length of the cell is much faster than the timescale of growth zone activation, therefore the two spatially separated growth zones compete for the same spatially uniform pool of substrate molecules.

Defining the parameters
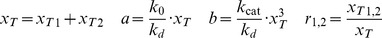
and introducing the change of variables



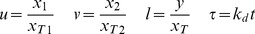
the system (1) can be non-dimensionalised as



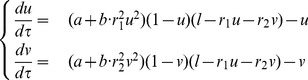
(2)Here 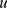, and 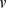 represent the proportion of active polarisome on the two cell ends, and 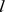 is a measure of the total substrate available. When 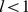 then the substrate present in the cell is less than what would be needed for the activation of all polarisomes, and when 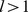 then the substrate is in excess and is sufficient to potentially fully activate the growth of both cell tips. The parameters 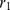 and 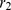 represent the proportion of total polarisomes on each tip relative the total amount of polarisome in the cell. Thus, by definition they satisfy: 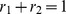, and reflect the “ability” of each cell tip to sequester the substrate.

Since the activation and de-activation reactions occur on a time-scale that is much faster than the characteristic time of cell growth, over which 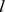 increases, for any value of 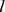 we can consider the system (2) at equilibrium. We are therefore interested in understanding how the equilibria of the system change as a function of the cell length 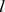 during the cell cycle. We will show that the results from such *bifurcation analysis* of the above system can be interpreted in agreement with the basic characteristics of the fission yeast growth patterns. We will also study how the bifurcation diagram changes when the model parameters are varied, and link these changes to altered growth phenotypes of various mutant strains. All the bifurcation diagrams have been created using G. B. Ermentrout's software XPPAUT [12].

## Results

The steady states of the system (2) correspond to the values of 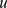 and 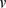 for which the rate of change of both components is equal to zero. An intuitive and effective way to understand how the steady states of the system depend on the cell length, is to follow the intersections of the nullclines 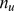 and 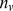, that are the curves on which the rate of change of either 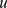 or 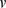 is zero, as 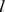 increases. The nullclines for the system (2) can be expressed analytically as:
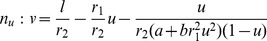
(3)

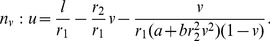
(4)


These expressions show that the shape of the nullclines depends on the parameters 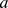, 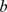, 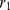 and 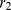, and 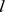 determines their position in the 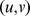 plane; specifically the nullcline 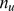 is shifted upwards when 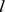 is increased, while at the same time the curve corresponding to 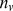 moves from left to the right.

### Symmetric cell

To understand the general behaviour of the system we first consider the case when 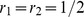, i.e. the total amount of polarisomes is symmetrically distributed between the two tips, thus they have the same potential for being activated. In this case the curves 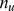 and 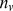 are mirror images of each other with respect to the line 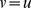. Consequently, the steady state solutions can be either symmetric, when the nullclines intersect on the line 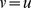, so that the growth activity of the two tips is the same (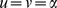); or a pair of asymmetric steady states, such as 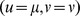 and 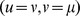.

**Figure 2:**
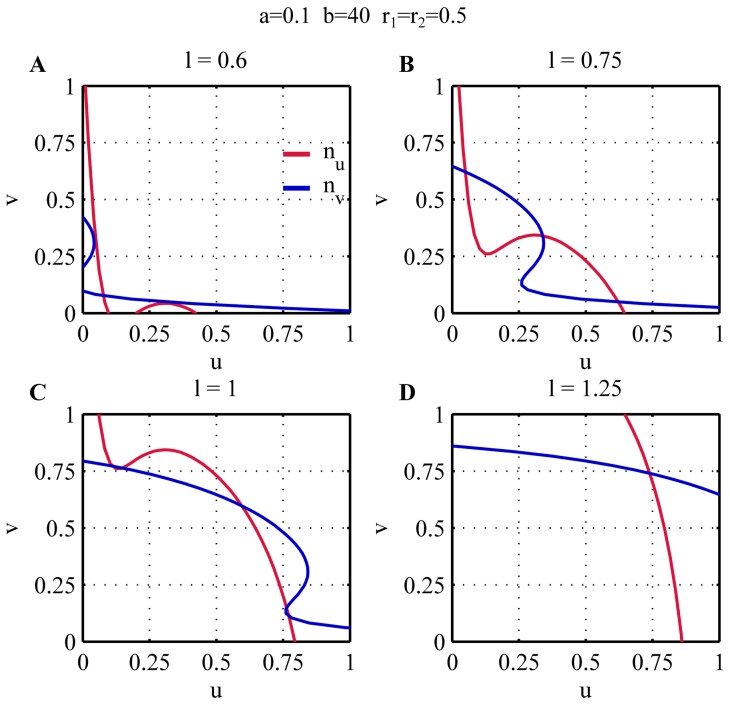
Nullclines of the system (2). The figure shows the nullclines on the 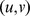 plane for different values of 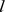, illustrating the appearance and disappearance of steady states when 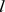 increases (**A–D**). For the simulations the parameters 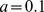, 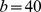, 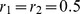 have been used.

**Figure 3:**
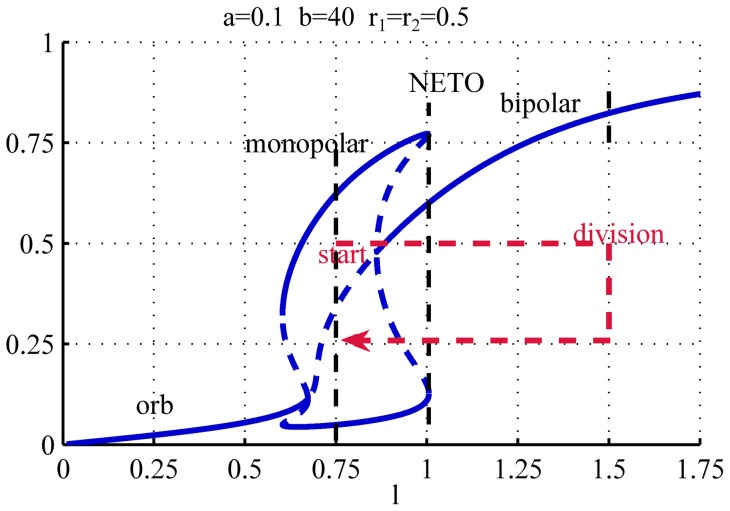
Bifurcation diagram representing the growth pattern of WT cells. For low values of 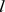 only one steady state exists in which both 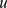 and 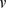 equilibrate at low values (*orb* branch). When 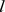 increases, the auto-catalytic activity on the tips overtakes the basal activation and 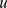 and 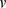 compete for 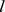. In this situation either 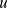 or 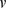 equilibrates at a high level while the other is at a low level (*monopolar* branch). As a result only one of the two tips is active and growing while the other stays inactive. As 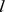 further increases there is enough substrate for the activation of both tips, and both 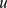 and 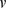 equilibrate at high values. This is the only possible solution at large 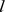 (*bipolar* branch). NETO can be interpreted as the point at which the asymmetric monopolar branch ceases to exist, after which both tips will be active. For the simulations the parameters 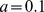, 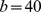, 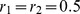 have been used.

Figure 2 shows the nullclines 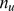 and 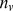 for different values of 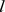 (**A–D**) for the case 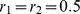, 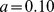, 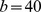. The corresponding bifurcation diagram is shown in Figure 3. Due to the symmetry of the system the bifurcation diagrams for 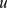 and 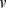 are exactly the same. For low values of 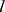 the amount of the substrate is not enough for the auto-catalytic activation to take over, and the system settles to a unique symmetric steady state in which both 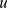 and 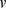 have the same low value (Figure 3A, 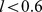). In this state none of the tips is active and the cell does not produce polarized growth. This kind of behaviour is analogous to the phenotype of orb mutant cells that are round-shaped (due to the lack of growth on the tips) and usually have a much smaller size than the wild type cells [13]. For this reason we refer to this solution branch on the bifurcation diagram as the *orb branch*.

As the value of 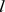 increases there is enough substrate for one of the tips to become activated, and the system undergoes a pitchfork bifurcation [14] (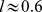, Figure 2A). From this value of 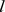 the system, in addition to the symmetric steady state (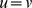), also has a pair of asymmetric equilibrium states, one for which 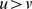 and one for which 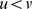 Figure 2B). The symmetric steady state is unstable (dashed line in Figure 3) meaning that although there is a potential equilibrium for these values of 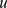 and 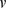, the slightest perturbation is enough to make the system abandon this state. Due to the intrinsic noise present in biological systems it is not possible to maintain such unstable state, thus only the pair of stable asymmetric solutions is relevant for our discussion. Thus for this range of values of 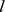 only one of the two tips is growing, and we refer to this branch of the bifurcation diagram as the *monopolar branch*. Note, that for the parameters corresponding to Figure 3 the pitchfork bifurcation at 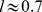 is sub-critical, and therefore there is a small overlap between the orb and monopolar regimes. The size and existence of this overlap, however depends on the parameters, that we will discuss later.

Increasing further the value of 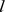 the system undergoes a second pitchfork bifurcation (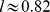) where the symmetric steady state becomes stable again, and a pair of new unstable asymmetric branches are created, that coexist with the monopolar branch. At this point there are multiple stable steady states present, and the system could equilibrate either on the asymmetric (monopolar) branch or on the symmetric (bipolar) one. In the case of a growing cell, when the symmetric stable branch appears, the solution is already on the stable monopolar branch, and therefore when 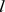 increases slowly it will follow this branch for as long as it exists. As 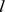 increases the pair of unstable asymmetric branches approach the stable monopolar branches until they collide and both disappear in saddle-node bifurcations at 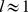 (Figure 2C). From this point the only steady state is the stable symmetric solution and the substrate is now sufficient to activate the second, previously inactive tip. This leads to a re-equilibration of the substrate, and the amount of active polarisome becomes the same on both tips. Thus, at the point when the monopolar branch ceases to exist the cell suddenly switches from monopolar to bipolar growth, that is consistent with the experimentally observed NETO. With the parameters used in Figure 3 if we assume that during the cell division cycle 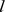 increases from 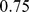 to 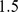, NETO happens approximately at 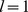 that corresponds to 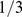 of the cell cycle (although this relies on the assumption that the growth in length is linear during the cycle, which is not entirely correct).

When the septation takes place each of the two daughter cells contain approximately half of the substrate 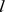, so that the activity of the tips in these cells repeats again the same growth pattern as shown in the bifurcation diagram. For 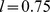 only the asymmetric equilibria are stable and the cells first start growing in a monopolar fashion. In the symmetric cell the steady states corresponding to either growing end have identical basins of attraction, and the growth site is selected by the initial condition corresponding to the state of the cell after division. It is observed that predominantly the old tip is activated first, that could be explained by having the substrate still at least partially bound to the old tip when the septation takes place. Thus after the division the old ends have an advantage over the new end to win the competition for the common substrate. The new end is activated later only upon reaching a threshold length, in agreement with the observed growth pattern.

### Parameter dependence

**Figure 4:**
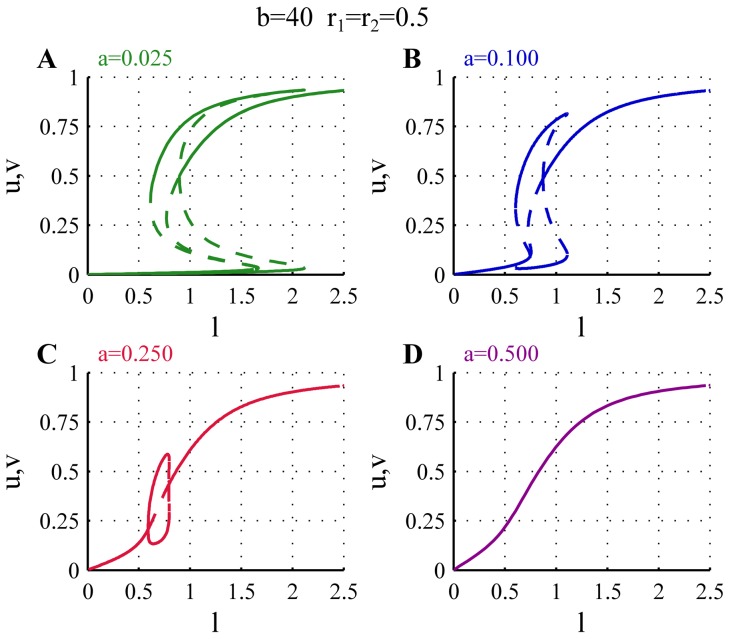
Effects of changing the parameter 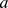 on the bifurcation diagram. The plot shows the bifurcation diagram for different values of 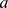. When 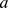 is lower than the value considered for the WT cells (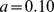, blue) the monopolar branches disappear at larger values of 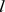 causing delayed or absent NETO (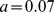, green). For higher 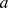 the monopolar branch is shorter (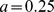, red), and eventually disappears completely (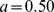, magenta). For all bifurcation diagrams 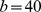, 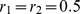.

As mentioned earlier the strength of the basal activation 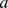 and of the auto-catalytic activation of the polarisomes 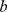 determine the shape of the nullclines. Therefore variations in these two parameters also produce changes in the bifurcation diagrams and influence the corresponding growth patterns. The effects of varying the parameter 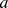 are shown in Figure 4. Reducing the value of 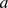 (Figure 4A), relative to the case discussed above, leads to a larger range of overlap between the orb, monopolar and bipolar regimes, although this does not affect the starting point of the monopolar branch. As the monopolar branch extends to larger values of 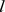, NETO is delayed in the cell cycle. Increasing the basal activation rate (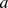) in general weakens the asymmetry of the monopolar branches, i.e. the amount of active polarisome on the active and inactive tips are less different, and the overlap between the monopolar and bipolar branches disappears. For larger values of 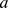 the asymmetric monopolar branches and the bifurcations disappear completely and only a single symmetric branch exists for all values of 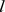 (magenta).

We can interpret these changes in the bifurcation diagram to predict the effects of mutations that influence the basal activation rate of the tips. Mutations that decrease the basal activation rate of the polarisome (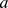) give rise to cells that exhibit delayed NETO. For low values of 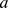 the increased overlap between the orb and the monopolar branches can also lead to mixed phenotypes in which a certain proportion of cells reach to the orb branch for which the tips do not extend, while the rest exhibit delayed NETO or only monopolar growth.

Mutations that increase the value of 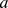 can create two different phenotypes. For moderate increase of 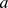 the cells still show a transition from monopolar to bipolar growth, but the activation of the monopolar growing tip is weaker, i.e. grows more slowly than in WT cells, and NETO is earlier Figure 4C, 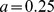). For larger values of 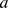 the growth is bipolar from the beginning of the cell cycle, since the bipolar branch extends below the initial length, but the speed of growth (assumed to be proportional to the polarisome activity) increases as the cell increases in size (Figure 4D, 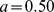).

**Figure 5:**
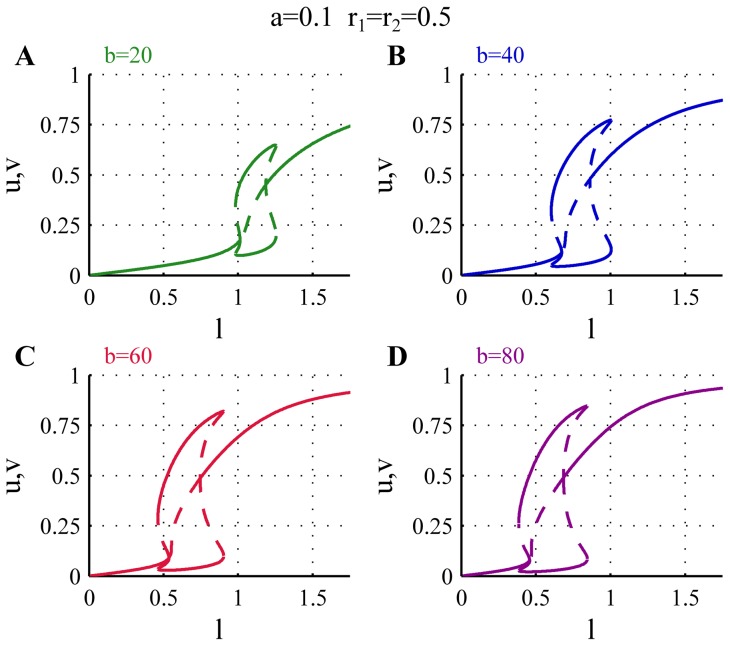
Effects of changing the parameter 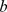 on the bifurcation diagram. The plot shows the bifurcation diagram for different values of 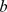. For low 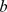 the bifurcations are shifted to larger values of 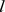 and the amount of active polarisome is lower (green). Increasing the value of 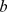 the bifurcations and monopolar branches move to lower values of 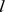 and there is an increase in the amount of active polarisome during both monopolar and bipolar growth (blue,red,magenta). For all bifurcation diagrams 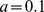, 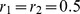.

Figure 5 shows the effects of changing the coefficient of autocatalytic activation of the tips. The main consequence of changing 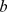 is that it shifts the bifurcations to lower or higher values of 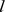. In addition it also has some effect on the strength of the asymmetry in the monopolar regime. For low values of 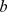 the monopolar branch appears for larger 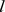 and the amount of active polarisome on the active monopolar branch is generally lower. Increasing 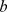 causes the monopolar branch to shift to lower values of 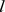, and as the autocatalytic activity is stronger the amount of active polarisome is slightly larger on the monopolar branch.

For mutations that affects the autocatalytic activation of the tips one can expect therefore the following phenotypes: Decreased value of 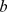 gives rise to cells that grow slower and display delayed NETO. If 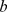 is too small the starting point lies on the orb state and the tips extend very poorly. On the other hand mutations corresponding to increased autocatalytic activity are expected to give rise to cells that grow faster and display early NETO.

**Figure 6:**
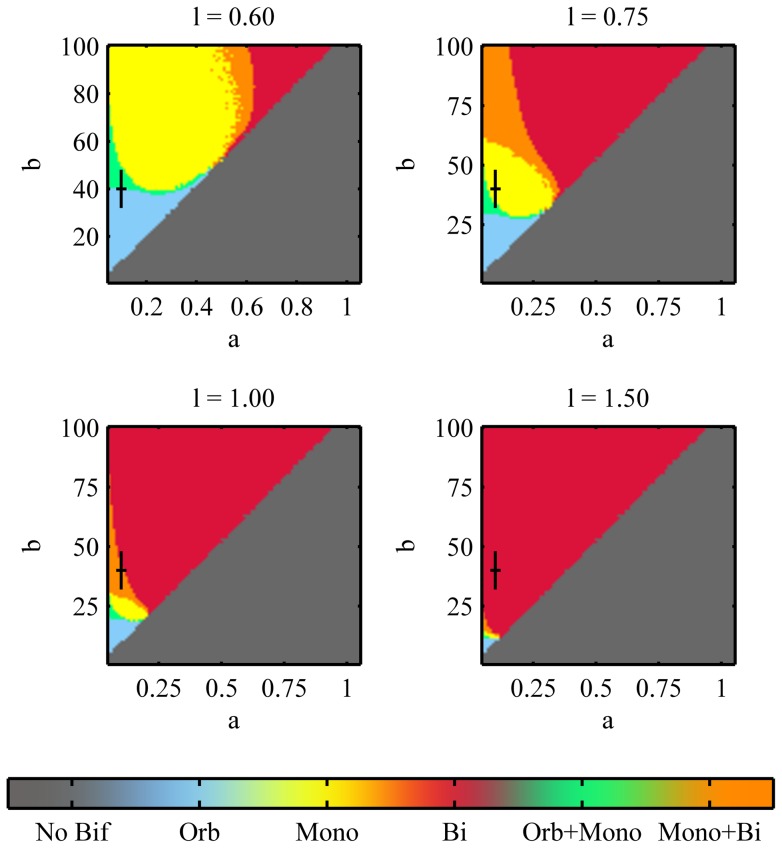
Effects of the parameters 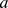 and 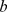 on the bifurcation diagram. The plots show the existence and overlap of the branches in the parameters space 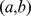 for different values of 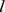. The grey area shows the values of 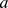 and 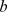 for which no bifurcations occur. The other regions show the region of existence of the various branches for different values of 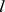. The black lines represent the values of 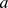 and 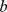 chosen for the WT cells 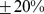.

The effects of variations in both 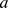 and 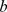 are generally more difficult to predict. Figure 6 shows how the various branches are arranged in the 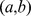 parameter space for four different values of 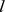. The “cross” corresponds to 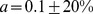 and 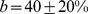, that is the values chosen to represent the WT cells 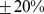. The grey area represents the values of 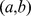 for which no bifurcations occur when varying 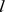 from 0.05 to 2.5. The boundary between the orange and the red areas represents where NETO takes place. In the previous examples we selected parameters representing WT for which 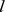 varies between 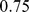 and 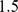 during the cell cycle and NETO takes place at 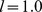. We have shown that the system with 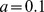 and 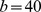 satisfies such constraints, but, as figure 6 shows, this choice is not unique. In fact any pair 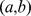 that lies on the line given by intersecting the monopolar region at 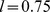 and the NETO curve at 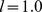 is a good candidate for the WT cells. The errorbars centred around our choice of values for the WT cells show that even in the presence of a 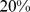 noise, at the starting point most cells would lie in the monopolar region, and that NETO would robustly take place around the value 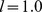 corresponding to one third of the cell cycle.

### Asymmetric cell tips

In the previous section we have considered the bifurcation diagrams and the corresponding growth phenotypes of fission yeast in the special case of symmetric cells when 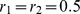 in equations (2). In this section we extend the results of our analysis to the case 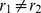, i.e. we study the effects of asymmetry in the distribution of the polarisomes on the tips.

The first consequence of 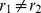 is that the nullclines 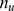 and 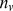 are no longer mirror images of each other with respect to the line 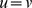. Due to the loss of symmetry the nullclines 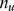 and 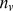 have in general different shapes and they move at different rates in the 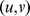 plane as 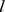 increases. The shift corresponding to an unitary increase of 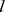 is equal to 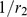 for 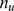 and 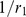 for 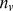, in the 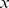 and 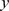 directions, respectively.

In general, since the two monopolar branches (one corresponding to high 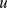-low 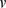, and the other with high 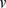-low 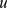) are not equivalent anymore, they undergo bifurcations at different values of the parameter 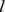, rather than simultaneously as in the symmetric case. This causes imperfect pitchfork bifurcations [14], [15] in which the pitchfork bifurcation degenerates into a pair of saddle node bifurcations that take place at slightly different values of the bifurcation parameter. Since this happens at both pitchfork bifurcations, in the ‘monopolar’ regime (i.e. intermediate values of 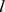) one stable branch together with the unstable branch forms a disconnected island in the bifurcation diagram, while the other stable mono polar branch remains fully connected to the orb and bipolar branches.

**Figure 7:**
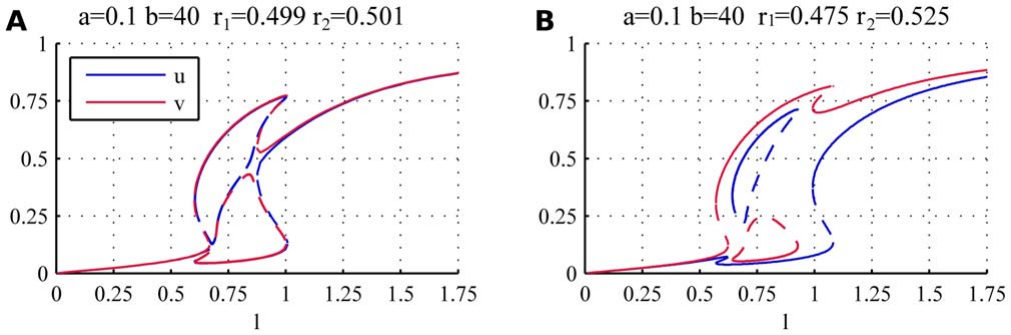
Bifurcation diagrams for weakly asymmetric system. The asymmetry causes the appearance of an island disconnected both from the orb and the bipolar branches (closed red and blue curves). The values 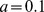, 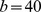, (**A**) 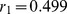, 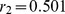 (**B**) 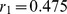, 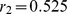 have been used.

Figure 7 shows bifurcation diagrams for two cases with weak asymmetry (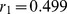, 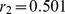 and 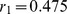, 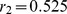) using the same parameters as for the symmetric WT cells (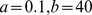). As in the symmetric case shown in Figure 3, we can identify three distinct regions for the activation of the tips. For low values of 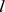 the activity of polarisomes on both tips is very low and as such the growth is inhibited; for high values of 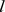 both tips are active, although 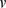 is favoured by the asymmetry and has slightly higher values than 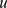. For intermediate values of 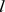 the growth is monopolar, i.e. either tip can be active while the other is inactive, but now the two stable monopolar branches are not equivalent. While one of the monopolar branches is connected to the orb and bipolar branches (high 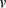 and low 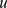 when 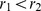 as in Figure 7), the remaining two branches (one stable and one unstable) form an island at intermediate values of 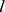 (closed red and blue curves in Figure 7), that terminates at both ends in saddle node bifurcations where the stable and unstable branches meet.

**Figure 8:**
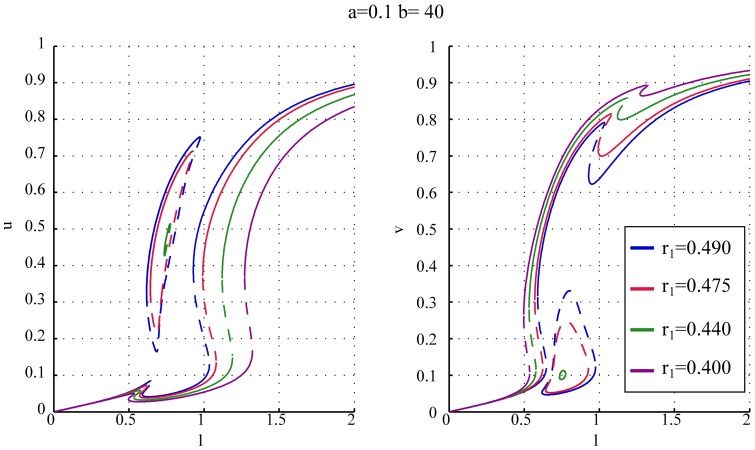
Individual bifurcation diagrams of 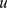 and 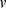 for various amount of asymmetry. The figure shows how the bifurcation diagrams of 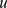 and 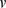 depend on the asymmetry in the system. For all the diagrams, the values 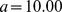 and 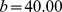 have been used.

Figure 8 shows the effect of increased asymmetry in the system where we plotted the steady states for 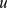 and 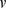 separately. For weak asymmetry (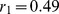, blue) although one of the monopolar branches is disconnected from the other branches, the regions in 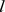 where they exist still overlap. As a consequence when the cell reaches the end of either monopolar branch (connected or disconnected) there is a direct transition to bipolar growth (NETO), as in the case of symmetric cells.

Increasing the asymmetry (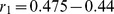) the stable monopolar branch that belongs to the island does not cover anymore the whole range of 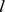 values between the orb and bipolar regimes. In this case two distinct patterns of growth are theoretically possible. When the monopolar growth is initiated on the connected branch (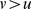 in Figure 8) then similarly to the symmetric case the growth is monopolar until the branch disappears and then NETO takes place followed by bipolar growth. The other pattern of growth is obtained when the monopolar growth is first initiated on the isolated stable branch that belongs to the island. When this branch disappears the cell cannot switch to bipolar growth yet, due to the gap between this monopolar and the bipolar branch. Instead the steady state first switches suddenly to the other monopolar branch and the cell continues monopolar growth on the opposite tip. Upon reaching the length for NETO the other end is re-activated and the growth proceeds on both tips.

Again we can assume that the presence of the substrate on the tips when the cell divides likely to select the old end to be activated first in the monopolar regime. In the case of an asymmetric cell we have two possibilities. When the old end has a larger amount of total polarisomes (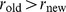) the cell follows the fully connected branch of the bifurcation diagram and proceeds with the standard growth pattern (i.e. switching directly from monopolar to bipolar growth) and the asymmetry does not have a significant effect. In the opposite case (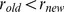) the monopolar growth initiated on the old end belongs to the stable branch of the island in the bifurcation diagram. In this case the bifurcation diagram predicts the modified growth pattern in which that transition to NETO is preceded by a sudden switch of polarization of the monopolar growth from the old end to the new end of the cell.

For high asymmetry (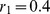, magenta) the island disappears and the only pattern of growth is the standard one: monopolar, NETO, bipolar. In this case one end inherits a very weak growth zone, therefore always the stronger tip is activated first regardless wether this is the new or old end of the cell and the anomalous growth pattern disappears.

### Three growth zones

In the previous sections we have considered only growth on the tips, here we extend our model by considering a third growth zone mislocalised at the lateral cortex. The polarisomes on the lateral cortex and the tips compete for the cytosolic substrate 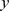 according to the same mechanism as previously described, resulting in the differential equations:
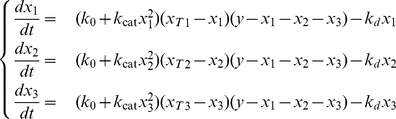
(5)


The two-component model (1) can be seen as a limit case of the system (5) obtained by setting 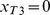.

As before it is convenient to non-dimensionalize the system (5) and consider
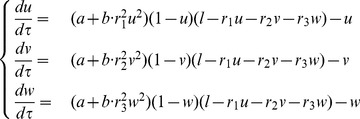
(6)where we have defined



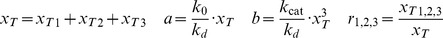
and introduced the variables:



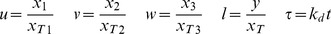



The study of the activation of growth zones can be carried out in a similar way to the cases discussed earlier. The main difference is that now the nullclines, i.e. the loci of points for which the rate of change of one of the components vanishes, are surfaces in the 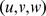 space described by the equations:
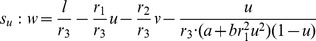
(7)

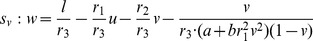
(8)

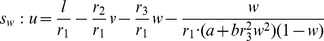
(9)


**Figure 9:**
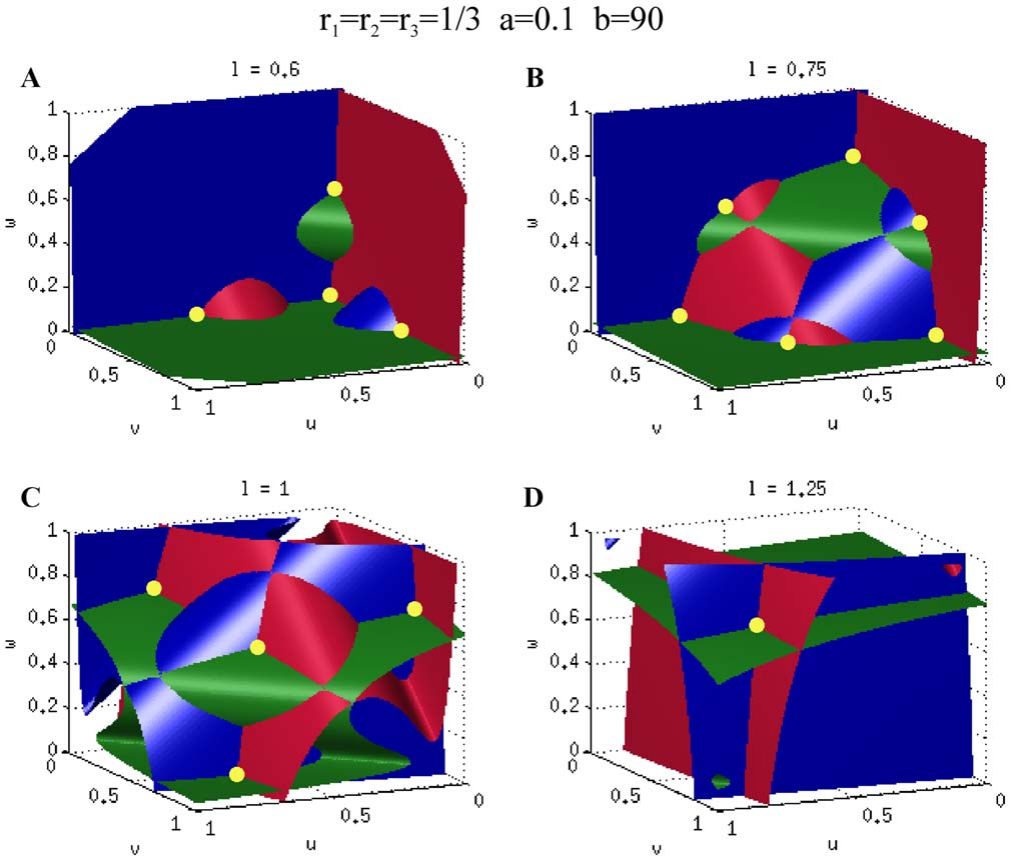
Nullcline surfaces. The parameter values are 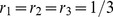, 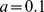 and 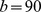. The stable equilibria, corresponding to the monopolar, bipolar and tripolar branches are marked by yellow dots in **A–D**.

The equilibria of the system can be identified as common intersections of all three nullcline surfaces. Similarly to the two-component case, the shape of the nullclines is entirely determined by the parameters 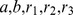 while the cell length parameter 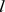 only moves the three surfaces in the directions of the 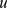, 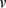 and 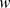 axis, respectively. The general situation for the symmetric case is shown in Figure 9 for the parameters 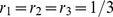, 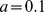 and 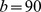. The Figure shows how the nullclines move in the 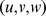 space as 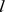 increases, and how they intersect creating and annihilating equilibrium points. Although the graphical representation is apparently more complicated, the behaviour of the system is similar to the two component model.

**Figure 10:**
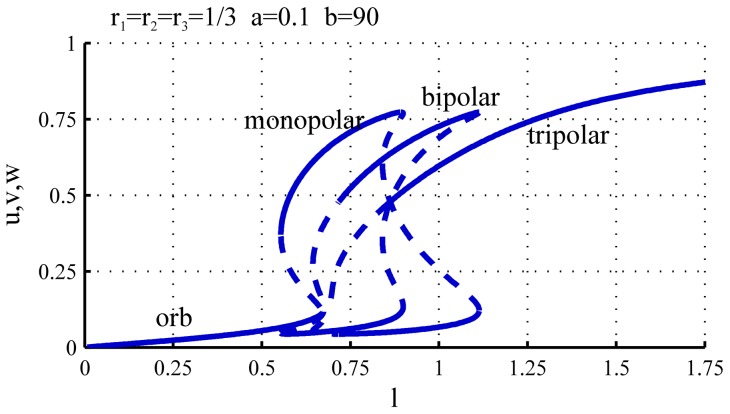
Bifurcation diagram. The parameter values are 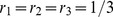, 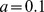 and 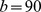. As 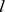 increases the system undergoes several bifurcations corresponding to the creation of monopolar, bipolar and tripolar branches.

For small values of 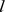 only one steady state exists, in which the growth at both tips and at the lateral cortex is inactive because the amount of substrate is not enough to activate either of them. Increasing 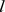 eventually provides sufficient substrate to activate one site of growth (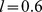 in Figure 9A). This active monopolar growth can be either at one of the tips or at the lateral cortex, similarly to the monopolar branches of the two component system. As 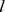 increases further, three stable symmetric bipolar branches are created (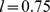,Figure 9B) that coexist with the monopolar branches. So the system can be in a state in which only one site of growth is highly active or in one with two sites active, with slightly weaker activation. Due to the symmetry all the possible configurations are present and the system has six stable steady states (yellow circles, Figure 9B). As the value of 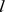 is increased the monopolar branches disappear, and at a higher value of 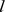 (Figure 9C) a new stable symmetric branch appears in which all three sites of growth are active. The region of existence of this tripolar solution overlaps with the bipolar branches. Further increasing 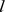 the bipolar branches disappear and there is only one steady state in which all growth zones are active (Figure 9D). The yellow circles in Figure 9A–D represent the stable solutions of the system. The corresponding bifurcation diagram is shown in Figure 10. Thus similarly to the two component system, increasing the amount of substrate, 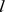, results in the creation of stable solutions with an increasing number of active growth zones, while solutions corresponding to fewer active zones disappear.

**Figure 11:**
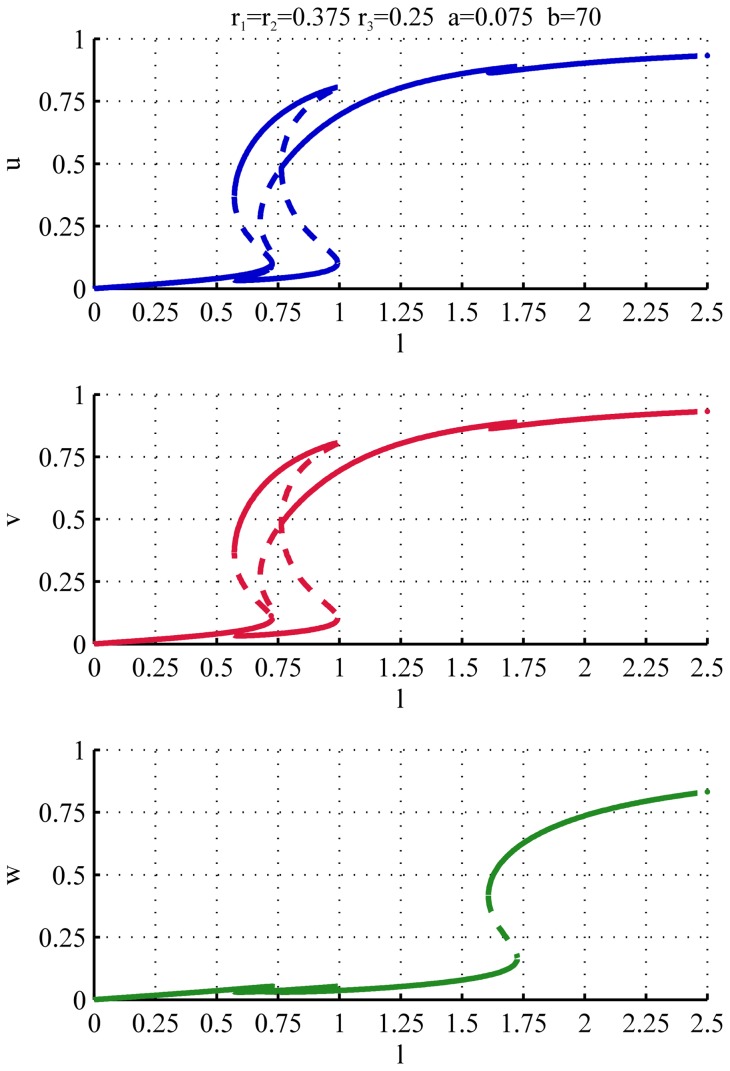
Two identical growth zones on the tips and a third weaker growth zone at the lateral cortex. The plots show the bifurcation diagram for the components 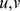 and 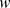. The tips follow the typical growth pattern of a symmetric cell, and the activation of the growth site at the lateral cortex is delayed to longer cell lengths. The parameters are 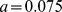, 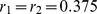, 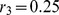, 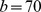.

Asymmetries in the system cause a change in the shape of the nullcline surfaces, and lead to a bias favouring growth zones with a greater amount of polarisome. This is shown for example in Figure 11 that displays the bifurcation diagram corresponding to the case in which the two tips have the same amount of polarisome, that is higher than at the lateral cortex (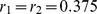, 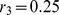). In this configuration of the parameters initially only 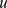 and 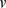 compete equally for the substrate while, due to the lower value of 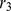, 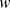 is excluded from the competition. Therefore, the bifurcation diagram for the tips is the same as in the symmetric two component model, and the value of 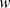 remains low. During the normal cell cycle progression, l is not high enough to activate growth at the lateral cortex. This can only be activated for higher concentrations of the substrate, as it is shown in Figure 11. This is in agreement with the phenotype of mutant cells (cdc25ts) whose entry into mitosis is inhibited and their microtubules are disrupted by a drug. These cells also establish a new growth zone at the middle of the cells but only activate it as a branch when their length becomes longer that the normal division size [3].

## Discussion

Eukaryotic cells exist in a huge range of shapes and sizes that ultimately determine their functions and ability to respond to the various external and internal stimuli [16]. Establishment and maintenance of polarity is an important feature of cells determining the correct assembly of cellular structures, regulating the cell functions [1] in a range of processes like embryogenesis and chemotaxis [17]. The fission yeast *Schizosaccharomyces pombe* and the budding yeast *Saccharomyces cerevisiae* have become popular to study cell morphogenesis and polarity regulation [18]. Budding yeast creates one bud in each cycle at a position controlled by historical landmark. It is an attractive organism to study polarity establishment by spontaneous symmetry breaking when the gene of the landmark protein is mutated [19].

S. pombe, in particular, has become an attractive organism for the study of cell polarity due to its characteristic shape and pattern of growth. The switch from monopolar to bipolar growth involves a polarity switch necessary for the activation of a new site of growth. By observing the pattern of growth of WT and mutants cells many proteins involved in the regulation and activation of the growth machinery have been identified. For example, microtubules have been shown to play a role in determining the sites of growth and in depositing the land-mark protein tea1 on the tips [20]; orb, tea and ban genes are involved in different ways in determining and maintaining the sites of growth, and in regulating the switch from monopolar to bipolar growth [6]; pom1p deposited on the tips throughout the cell cycle is important for symmetric division [21] and for entering mitosis at the right length [22]–[24]; bud6p, cdc42 and for3 are required for actin nucleation on the tips [10]. Recent experimental evidence has shown that microtubules, although necessary for the correct positioning of the growth machinery and shape maintenance, they are not required for the establishment of a new site of growth per se [3]. Moreover the study of depolarized spheroplasts has shown that fission yeast has the ability to re-establish the rod shape by activating sites of growth, whose width is genetically determined, in a way similar to the creation of sites of growth of budding yeast [25].

The increasing biochemical knowledge about polarity regulation created a demand for modelling in order to identify the underlying design principles. Several models investigating different aspects of cell polarity have been proposed (see [26] for a comprehensive review). For example, the growth pattern of fission yeast polarity mutants has been analyzed by Boolean network in order to determine the underlying influence diagram [27]. The role of surface tension at the membrane in recruiting the growth machinery, NETO and positioning the septum ring has also been investigated [28]. The mechanism of polarity establishment is most intensively analyzed in budding yeast. Bud formation has been shown to be due to a Turing-like mechanism in which one of the loci of polarization wins the competition for Cdc42 activating the growth of a single bud per cell cycle [29]. Reaction-diffusion mechanisms based on actin diffusion and polymerization have also been proposed to be responsible for the fission yeast growth pattern [11]. In this model, the actin polymers in the growing tips are competing for the rapidly diffusing actin monomers and polymerize them autocatalytically. The principle of competition and positive feedback has been also included in a recent model to describe the observed oscillations of active Cdc42 at cell tips of fissions yeast [9]. To explain the oscillatory behavior of active Cdc42 the model was extended by a time-delayed negative feedback loop as well. However, it appears that the presence of oscillations do not influence the characteristics of the transition from monopolar to bipolar growth, and their biological role remains unclear.

In our work we focus our attention on the mechanism that allows for the activation of a site of growth at a specific length considering the global role of the proteins in the polarisome, rather than studying the temporal dynamics of a specific protein. To do this we have completely separated the problem of localising the growth machinery and the one of its activation. Our model shows that the pattern of growth of fission yeast and its length-sensing mechanism required for NETO can be explained just by considering auto-catalytic activation of the polarisomes and the competition for a common substrate. Moreover changes in the parameters can be linked to the patterns of growth of various different mutant cell types observed experimentally, providing clues for the role of the proteins in the polarisome complex.

In WT cells the majority of the population switches from monopolar to bipolar growth. Such growth phenotype can be easily linked to the bifurcation diagram shown in ??fig:odend_bif n which the competition for the substrate selects one of the two tips to be activated, and the other is activated only upon reaching a threshold length. After division the previously active old end creates a bias so that the initial state has a higher probability to belong to the basin of attraction of the active old end while the new end stays initially inactive. On the other hand we have shown that strongly asymmetric cell division may lead to growth patterns where the new end is activated before the old end; some evidence for these behaviours can be found in the experimental data presented in [9]. However, depolarizing the cells disrupting the actin network when the cell is growing monopolarly causes a switch from monopolar to bipolar growth [30] providing evidence for the overlap of the monopolar and bipolar branches, as noted in [11].

The growth pattern of several mutants can also be linked to bifurcation diagrams of the two-component model. For example, tea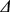, bud6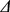 and pom1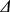 mutants display no NETO [5], that can be explained in our model by a lower value of the parameter 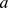, suggesting that these proteins play a major role in the basal activation of the polarisome rather than in its auto-catalytic activation. The behaviour of some of these mutants differ in the selection of the end that starts growing first after division. For example bud6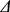 mutant will grow in a monopolar fashion extending only the old ends. Even though one of the old ends has never grown before, in our model a small amount of active polarisome is always present on the inactive tip, thus favouring the activation of the old end of the daughter cell over the newly formed one. In tea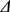 mutants after division one of the daughter cells continues growing from the previously growing end, while the other grows on the new end. A possible explanation of the growth initiation at the new end is that in the absence of tea proteins, the polarisome is not delivered to the tips so that residual actin patches from the division ring, on the new end may confer a small advantage in the competitive growth activation.

for3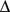 and rga4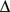 cells show defects in the diameter, but also a very peculiar pattern of growth. After division the daughter cells show two different patterns of growth. One of the daughter cells starts monopolar growth on the old end and displays no NETO, while the other has a bipolar growth pattern. This can be explained based on our model by a bifurcation diagram where the monopolar and bipolar branches co-exist over the whole range of cell lengths corresponding to the cell cycle (e.g. due to reduced basal activation rate, 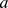). A cell that follows the normal growth pattern initiates monopolar growth of both daughter cells that extends until cell division. In the following generation, however, the cell divides into a daughter with a previously active old end, inducing monopolar growth, and another daughter cell whose neither end has been activated previously. Therefore, the initial state of this symmetric daughter cell falls into the basin of attraction of the symmetric bipolar branch, and the cell start bipolar growth from the beginning of the cell cycle. The daughter cells of the bipolar cell will have again an active old end follow monopolar growth. Thus, in this case both bipolar and monopolar growth patterns persist indefinitely in certain proportion in the population, as observed in the experiments.

undergoes bipolar growth, while the other cell grows monopolarly. One possible explanation of this is to assume that in for3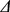 cells, the scar of growth somehow helps the establishment of the polarisomes on the tips. In this way the cell that has inherited the scar is approximately symmetrical and follows the standard pattern of growth, while the daughter cell that has no scar is strongly asymmetric in favour of the old end that wins the competition for the substrate through the whole cell cycle.

Our analysis also predicts certain growth patterns that have not been described so far in experiments. This is for example the case of moderately asymmetric division of polarisomes, that leads to a growth pattern where a certain proportion of cells switches the active ends during monopolar growth before the start of bipolar growth. Although, such growth dynamics has not been described, this type of transitions may be linked to the observed distribution of growth patterns in certain experiments.

The three component model might be useful to understand the behaviour of certain branched cells. For example experiments conducted on cdc25 cells locked in G2 phase, have shown that bipolar growing cells can activate a third site of growth when they reach a length that is almost double of the division length of WT cells [3]. This suggests that the third site of growth that we take into account in our model is strongly disadvantaged with respect to the tips in the competition for the substrate. A proportion of teal-3 cdc11–119 mutant cells however show several branches that are actively growing [6]. The presented model could be extended to describe such exotic cell shapes by considering a system with many competing growth zones. However, as the cell length increases the spatial aspect of the problem may became important, and the diffusive transport rates would also need to be taken into consideration in a spatially extended version of the model.

The proposed growth activation model allowed us to formulate possible dynamical mechanisms that can explain the observed growth dynamics present in wild type and mutant cells. Further experiments could test the proposed mechanisms and aim to identify the key molecular components and protein interaction network responsible for the activation of growth zones in fission yeast cells. This could be done by inhibiting or over-expressing certain proteins that are expected to influence the activation of growth zones. Their role then could be identified by analogy with the model parameters that produce the type of qualitative change in the bifurcation diagram that is consistent with the experimentally observed growth pattern (delayed or early NETO, no NETO, orb, mixed phenotypes etc.). Further information could be obtained by statistical analysis of the distribution of multiple growth patterns within a population and analysing correlations with asymmetric cell division.

NETO, using mass-spectrometry technology might be a way to find possible candidates for the common substrate 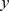. Also dense time-course measurements similar to the one carried in [9] of the other proteins co-localizing on the tips, would allow to determine the presence of other regulatory mechanisms, like negative feedbacks, that could be used to construct a refined mechanistic model of the regulation of polarised growth.
